# The *Kampo* Medicine Yokukansan Decreases MicroRNA-18 Expression and Recovers Glucocorticoid Receptors Protein Expression in the Hypothalamus of Stressed Mice

**DOI:** 10.1155/2015/797280

**Published:** 2015-05-14

**Authors:** Shoko Shimizu, Takashi Tanaka, Takashi Takeda, Masaya Tohyama, Shingo Miyata

**Affiliations:** ^1^Division of Molecular Brain Science, Research Institute of Traditional Asian Medicine, Kinki University, Osaka-sayama, Osaka 589-8511, Japan; ^2^Division of Women Medicine, Research Institute of Traditional Asian Medicine, Kinki University, Osaka-sayama, Osaka 589-8511, Japan; ^3^Osaka Prefectural Hospital Organization, Osaka 558-8558, Japan

## Abstract

It is well known that glucocorticoid receptor (GR) signaling regulates the hypothalamic-pituitary-adrenal (HPA) axis, and GR expression level is associated with HPA axis activity. Recent studies revealed that microRNA- (miR-) 18 and/or 124a are candidate negative regulators of GR in the brain. The *Kampo* medicine Yokukansan (YKS) can affect psychological symptoms such as depression and anxiety that are associated with stress responses. In this study, we evaluated the effect of YKS on miR-18 and 124a and GR levels in mice exposed to stress. We found that YKS pretreatment normalized elevated plasma corticosterone levels in stress-exposed mice. In addition, GR mRNA levels were downregulated in the brain following stress exposure. While miR-124a expression levels were not altered in the hypothalamus of stress-exposed mice, miR-18 levels decreased in the hypothalamus of YKS-pretreated mice after stress exposure. Finally, GR protein levels in the paraventricular nucleus (PVN) of the hypothalamus after stress exposure recovered in YKS-pretreated mice. Collectively, these data suggest that YKS normalizes GR protein levels by regulating miR-18 expression in the hypothalamus, thus normalizing HPA axis activity following stress exposure.

## 1. Introduction

Hypothalamic-pituitary-adrenal (HPA) axis activation is one of the key responses following physiological stress [1–5]. Corticosteroids provide negative feedback to the HPA system at the level of the hippocampus, hypothalamus, and pituitary gland by activating glucocorticoid receptors (GRs) [6–9]. At rest, the mineralocorticoid receptor has a high affinity for corticosterone [10]. However, GR has a low affinity for corticosterone and is rarely activated in resting conditions. Thus, GR expression is a crucial indicator of stress response indicator [10–14]. However, little is known about the molecular mechanisms regulating GR expression in the hypothalamus.

Yokukansan (YKS) (Tsumura, TJ-54) is a traditional Japanese medicine administered to patients who show symptoms such as nervousness, short-tempered behavior, irritability, sleeplessness, eyelid twitching, and limb shaking [15]. Furthermore, YKS ameliorates behavioral and psychological symptoms such as aggression, anxiety, and depression in patients with Alzheimer's disease and other forms of dementia. However, the mechanism underlying YKS-mediated attenuation of psychological symptoms and several stress-response behaviors is unknown.

MicroRNAs (miRs) are noncoding RNAs that inhibit the translation and/or decrease the stability of their target mRNAs, ultimately decreasing their protein expression levels [16, 17]. Recent studies indicate that miRs are involved in various functions such as embryonic development, differentiation, and neural plasticity [18–21]. Interestingly, miR-18 and/or miR-124a are candidate negative regulators of GRs in the brain [22]. Given YKS's stress-modifying capabilities, we hypothesized that it could exert its effect by impacting the HPA axis, and more specifically, by regulating GR expression in the hypothalamus. In the present study, we demonstrate that YKS affects hypothalamic miR-18 expression levels in stress-exposed mice and investigate GR protein expression level in the paraventricular nucleus (PVN) of the hypothalamus in YKS-pretreated and stress-exposed mice.

## 2. Materials and Methods

### 2.1. Ethics Statement

All animal care and handling procedures were approved by the Institutional Animal Care and Use Committee of Kinki University (no. KAME-24-021), and the Guiding Principles for the Care and Use of Laboratory Animals and the United States National Institutes of Health Guide for the Care and Use of Laboratory Animals were followed.

### 2.2. Animals

Adult male C57/BL6 mice weighing 25–35 g were obtained at 11 weeks of age from Japan SLC, Inc. (Hamamatsu, Japan). Three mice per cage were housed in a temperature- (22 ± 2°C), humidity- (55 ± 10%), and light- (12-h light/dark schedule, lights on at 07:00 and off at 19:00 h) controlled environment and were fed laboratory food and water* ad libitum*. The animals were allowed to adjust to the environment for 1 week before the experiments were performed. A randomized design was used to assign the mice to one of three groups (*n* = 12 in each group): no-stressed control, stress-exposed, and YKS-pretreated stress-exposed.

### 2.3. Stress Exposure

Stress exposure was performed as previously described [23]. Briefly, the mice were placed in a 50 mL conical polypropylene centrifuge tube and immersed vertically to the level of the xiphoid process in a water bath at 23°C for 2 h. In our preliminary experiments, chronic stress exposure did not induce gastric ulcer formation. Control mice were removed from their home cages and placed in new breeding cages for 2 h. Immediately after the end of testing, mice were anesthetized with sodium pentobarbital (30 mg/kg) and perfused transcardially with 4% paraformaldehyde (PFA) in 0.1 M phosphate buffer.

### 2.4. Drug Administration

YKS is composed of seven dried medicinal herbs: 19.5% Atractylodes lancea rhizome (ALR; rhizome of* Atractylodes lancea* De Candolle, Compositae), 19.5% Poria sclerotium (PS; sclerotium of* Poria cocos* Wolf, Polyporaceae), 14.6% Cnidium rhizome (CR; rhizome of* Cnidium officinale* Makino, Umbelliferae), 14.6% Japanese Angelica root (JAR; root of* Angelica acutiloba* Kitagawa, Umbelliferae), 9.8% Bupleurum root (BR; root of* Bupleurum falcatum* Linné, Umbelliferae), 7.3% Glycyrrhiza (GR; root and stolon of* Glycyrrhiza uralensis* Fisher, Leguminosae), and 14.6% Uncaria hook (UH; hook of* Uncaria rhynchophilla* Miquel, Rubiaceae) [24]. The seven medical herbs were extracted with purified water at 95°C for 1 h, and the extraction solution was separated from the insoluble waste and concentrated by removing water under reduced pressure. Spray-drying was performed to produce dried extract powder. The dry powdered YKS extracts used in the present study were supplied by Tsumura & Co. (Tokyo, Japan) and dissolved in distilled saline. The mice (*n* = 12 in each group) received oral YKS (1.0 g/kg of body weight) or saline 1 h before stress exposure.

### 2.5. Measurement of Plasma Corticosterone Levels

After mice were deeply anesthetized at the end of the stress experiment, and blood samples were collected into tubes containing heparin between 11:00 and 13:00 h by cardiac puncture. The tubes were immediately placed on ice and then centrifuged at 1,000 g for 15 min at 4°C. Plasma was stored at −80°C prior to the enzyme immunoassays. Plasma corticosterone levels were determined in duplicate using an AssayMax Corticosterone enzyme-linked immunosorbent assay kit (Assaypro, St. Charles, MO, USA).

### 2.6. Reverse Transcriptase Reaction and Real-Time Polymerase Chain Reaction (PCR)

Total RNA was prepared from the hippocampus and hypothalamus of mice using ISOGEN II (NipponGene, Toyama, Japan) according to the manufacturer's instructions. The total RNA extract was reverse transcribed by using oligo(dT)12–18 primers and ReverTra Ace qPCR RT Master Mix with gDNA Remover (Toyobo Life Science, Tokyo, Japan) according to the manufacturer's instructions. Real-time PCR was performed using an ABI PRISM 7900HT Sequence Detection System with the THUNDERBIRD qPCR Mix (Toyobo Life Science). To quantify the expression levels of GR, the following primers were used: GR forward primer, 5′-GTACCTCTGGAGGACAGATGTA-3′ (complement of bases 1025–1044); GR reverse primer, 5′-GCACCTATTCCAGTTTTCAG-3′ (complement of bases 1133–1152). Glyceraldehyde 3-phosphate dehydrogenase (GAPDH) forward primer 5′-GTGTTCCTACCCCCAATGTG-3′ and GAPDH reverse primer 5′AGGAGACAACCTGGTCCTCA-3′ were used as the internal controls. SYBR Green I fluorescence from the double-stranded PCR products was measured according to the manufacturer's instructions (Toyobo Life Science).

### 2.7. Quantification of miR-18 and 124a

Total microRNA was prepared from the hippocampus and hypothalamus of mice by using ISOGEN II (NipponGene) according to the provided instructions. The microRNA extract was reverse transcribed with the TaqMan MicroRNA Reverse Transcription Kit and TaqMan MicroRNA Assays has-miR-18a and mmr-miR-124a (Life Technologies, Inc., Carlsbad, CA, USA) as described by the manufacturer. Real-time PCR was performed using an ABI PRISM 7900HT Sequence Detection System with TaqMan MicroRNA Assays has-miR-18a (Life Technologies, Inc.). The comparative CT method (ΔΔCt) was used to quantify the relative expression levels of miR-18 and 124a according to the manufacturer's instructions.

### 2.8. Immunohistochemistry

Immunohistochemical analysis was performed as described previously [23, 25]. The brain sections (10 to 12 sections of the PVN region from each mouse) were immersed in guinea pig anticorticotropin-releasing factor (CRF) (1 : 500; Bachem Inc., Torrance, CA, USA) or rabbit anti-GR (1 : 100; Santa Cruz Biotechnology Inc., Santa Cruz, CA, USA) at 4°C for 24 h. The sections were then rinsed with phosphate-buffered saline (PBS) for 60 min and incubated at room temperature for 1 h with Alexa Fluor 568-conjugated goat anti-rabbit and Alexa Fluor 488-conjugated goat anti-guinea pig IgG antibody (Life Technologies, Inc.) at a dilution of 1 : 500 in PBS. Finally, the sections were washed with PBS for 1 h and mounted on slides using PermaFluor. Confocal microscopy (LSM-510 META) was performed with 20, 40, and 60x objective lenses (Carl Zeiss, Oberkochen, Germany).

### 2.9. Statistical Analyses

The statistical significances of differences were evaluated by two-tailed Student's* t*-tests unless otherwise mentioned, and differences were considered significant at *P* < 0.05. All data are presented as the mean ± SEM, and the number of experiments is indicated.

## 3. Results

### 3.1. YKS Normalizes Stress-Upregulated Plasma Corticosterone Levels

The HPA axis is reliably activated by stress exposure, and it is well known that this increases plasma corticosterone levels for up to 6 h after stress exposure [14, 23, 26]. YKS was previously reported to significantly affect mouse behavior just 1 h after a single oral dose of YKS, and several components of YKS cross the blood-brain barrier and function for at least 12 h after oral administration [27–30]. Thus, our study conditions were appropriate for studying stress responses and the effects of YKS.

**Figure 1:**
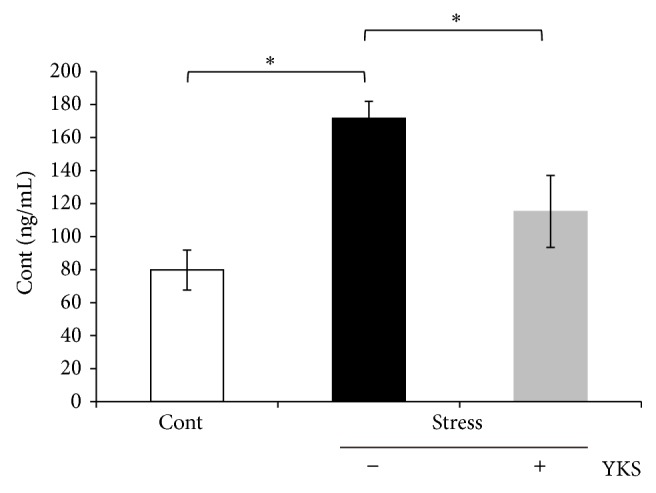
Acute stress upregulates HPA axis activity and alters plasma corticosterone levels. Results are the means ± SEMs of at least four independent experiments. Cont: control mice; Stress (YKS−): acute stress-exposed mice; Stress (YKS+): 1.0 g/kg YKS pretreatment and stress-exposed mice; ^∗^ *P* < 0.05, Student's* t*-test.

To evaluate HPA axis activation in our mouse model of acute stress, we first measured plasma corticosterone levels in control and stress-exposed mice. The stress-exposed mice exhibited upregulated plasma corticosterone levels (Figure 1, Stress-YKS). However, YKS pretreatment of stress-exposed mice normalized plasma corticosterone to levels comparable to those measured in control mice (Figure 1, Stress + YKS). We previously reported that Sgk1 expression in the corpus callosum was upregulated after acute stress [23]. However, this stress response was not detected in YKS pretreatment mice.

### 3.2. Stress Exposure Decreases GR mRNA Expression in the Hippocampus and Hypothalamus

**Figure 2:**
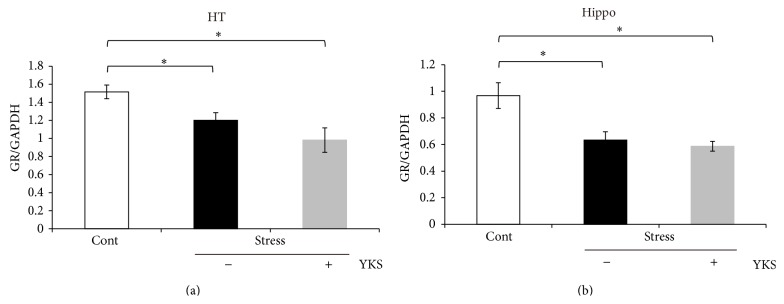
Real-time PCR analysis of GR mRNA expression in the hippocampus (a) and hypothalamus (b) after acute stress exposure. Results are the means ± SEMs of at least four independent experiments. Cont: control mice; Stress (YKS−): acute stress-exposed mice; Stress (YKS+): 1.0 g/kg YKS pretreatment and stress-exposed mice; ^∗^ *P* < 0.05, Student's* t*-test.

GR expression level is likely associated with HPA axis activity [14]. Thus, we examined GR mRNA levels in the hippocampus and hypothalamus after stress exposure. GR mRNA expression was decreased in both regions, and YKS pretreatment did not affect levels in stress-exposed mice (Figure 2, Stress + YKS). We further examined GR mRNA levels in YKS-pretreated and unstressed mice. GR mRNA levels were not significantly different between the two groups.

### 3.3. YKS Reduces miR-18 Expression in the Hypothalamus after Stress Exposure

**Figure 3:**
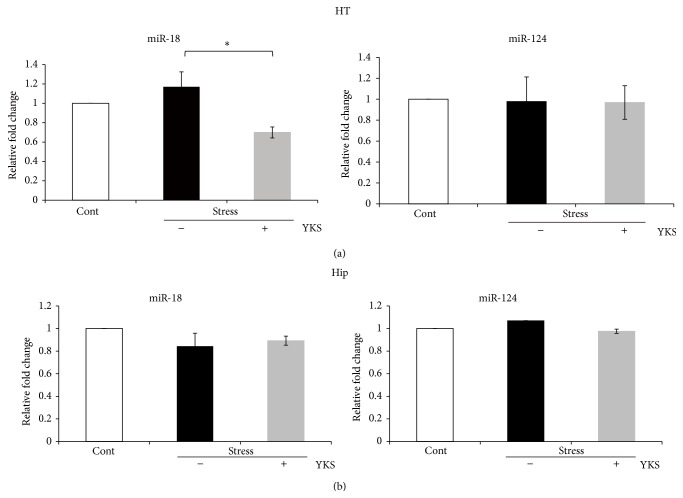
Real-time PCR analysis of miR-18 (a) and miR-124a (b) expression in the hippocampus and hypothalamus after acute stress exposure. Results are the means ± SEMs of at least four independent experiments. Cont: control mice; Stress (YKS−): acute stress-exposed mice; Stress (YKS+): 1.0 g/kg YKS pretreatment and stress-exposed mice; ^∗^ *P* < 0.05, Student's* t*-test.

The above findings indicated that YKS does not directly regulate GR mRNA. Next, we determined if YKS is involved in the posttranscriptional regulation of GR protein levels by evaluating the effect of YKS on miR-18 and 124a expression in the hippocampus and the hypothalamus following stress exposure. YKS pretreatment only reduced miR-18 expression in the hypothalamus (Figures 3(a) and 3(b)). miR-124a expression levels were unchanged in both regions after stress exposure (Figures 3(a) and 3(b)). These findings indicate that YKS regulates miR-18 expression in the hypothalamus but has no effect on GR mRNA or miR-124a after stress exposure.

### 3.4. YKS Normalizes GR Protein Level in the PVN of the Hypothalamus after Stress Exposure

We found that YKS pretreatment reduced miR-18 expression in the hypothalamus (Figure 3(a)). To obtain definitive evidence that this downregulation of miR-18 reduced inhibition of GR protein translation, we analyzed GR protein expression levels in the PVN of the hypothalamus.

**Figure 4:**
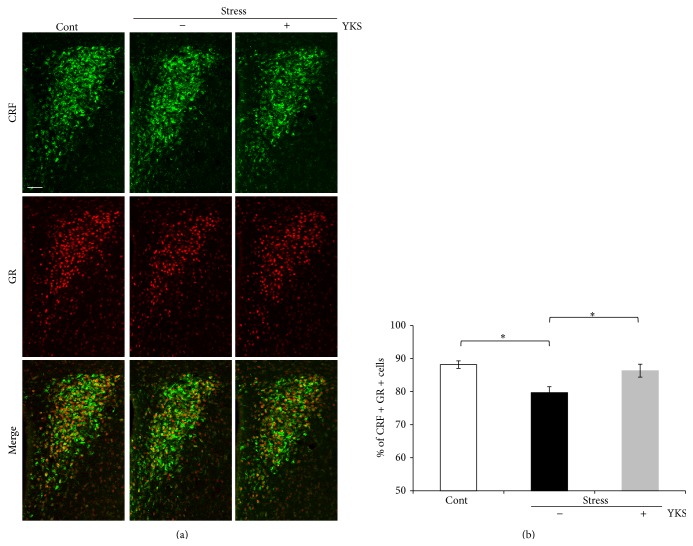
GR protein expression in the PVN of the hypothalamus. (a) Immunohistochemical analysis of CRF and GR in the PVN of the hypothalamus 2 h after the end of stress exposure. Scale bar, 50 *μ*m. (b) Measurements of merged cell numbers in the PVN region. Results are the means ± SEMs of at least four independent experiments. The cell counts were Cont, 1086; Stress, 804, and Stress + YKS, 1141. Cont: control mice; Stress (YKS−): acute stress-exposed mice; Stress (YKS+): 1.0 g/kg YKS pretreatment and stress-exposed mice; ^∗^ *P* < 0.05, Student's* t*-test.

It is well known that GR protein and CRF are expressed in the parvocellular division of the PVN of the hypothalamus and that CRF is secreted in response to stress [31, 32]. We first located the parvocellular division of the PVN by examining CRF expression (Figure 4(a)). CRF immunoreactivity (IR) was slightly upregulated in response to stress exposure (Figure 4(a)). However, YKS pretreatment suppressed this increase in CRF-IR in the PVN after stress exposure (Figure 4(a)). Furthermore, the number of CRF-IR cells in the PVN was not changed by stress exposure with or without YKS pretreatment (Figure 4(a)).

We found that stress exposure decreased the number of GR-IR cells, but this effect was blocked by YKS pretreatment (Figures 4(a) and 4(b)). These results suggest that YKS reduces miR-18 expression and increases GR protein expression by downregulating the inhibitory effects of GR translation in the PVN of the hypothalamus after stress exposure.

## 4. Discussion

YKS has been used to treat the behavioral and psychological symptoms of dementia (BPSD). It is also administered to patients with schizophrenia, certain pain diseases, delirium and dementias, and Parkinson's disease [33, 34]. Since YKS was found to improve BPSD, especially psychological symptoms such as anxiety, depression, and apathy in patients with several types of dementias, we initially examined HPA axis activity by measuring plasma corticosterone levels. We found that YKS regulated HPA axis activity by decreasing corticosterone levels in stress-exposed mice.

In the present study, we provide the first evidence that YKS downregulates miR-18 expression in the hypothalamus after stress exposure and ultimately normalizes GR protein levels in the PVN, thus affecting HPA axis activity.

It is well known that GR protein regulation is involved in HPA axis activity and reflects circulating plasma corticosterone levels and that corticosterone activates GRs in the hypothalamus [6–9]. Furthermore, GR expression level is regulated by several transcriptional factors and splicing regulators [35–37]. Recently, other regulatory mechanisms controlling GR expression levels have been reported including mRNA degradation, specific transcription factors activation, and regulation of microRNA expression [22, 38, 39]. We found that oral YKS administration did not affect GR mRNA levels, and we then focused on the posttranscriptional regulation of GR expression levels by microRNAs. In the brain, miR-18 and/or miR-124a posttranscriptionally regulate GR protein expression [22, 40, 41]. In our stress model, miR-124a expression was unchanged in the hypothalamus and hippocampus, whereas miR-18 expression was only downregulated in the hypothalamus of stress-exposed mice with YKS pretreatment. The finding of miR-18 reduction in the hypothalamus is in good agreement with a previous report [40]. Therefore, miR-18 expression may crucially control GR protein expression in the hypothalamus after stress exposure (Figures 3 and 4).

Previous studies reported that GR is expressed in CRF-positive neurons of the PVN region [31, 32]. While the number of GR-expressing cells decreased after stress exposure, we found that stress-exposed animals that received YKS pretreatment had similar numbers of cells compared to control animals (Figure 4). However, little is known about the molecular mechanisms by which YKS regulates GR expression in the hypothalamus. Previous studies reported that the HPA axis is regulated by several neurotransmitters including serotonin, dopamine, and norepinephrine [42, 43]. YKS has a partial agonistic effect on serotonin 1A receptor (5-HT1A) and suppresses 5-HT2A receptor activity [44–47]. Another report described GRs as an indirect target of antidepressants that affect serotonin neurotransmission [48]. However, YKS administration was not found to alter 5-HT2A receptor expression in the hypothalamus [45]. Based on our results and these earlier reports, posttranscriptional GR regulation in the hypothalamus by YKS is likely unrelated to serotonin neurotransmission.

YKS is a mixture with seven components, and it is important to determine which components affect stress responses in the HPA axis and posttranscriptional GR regulation in the hypothalamus. Several components of YKS (e.g., geissoschizine methyl ether and glycyrrhetinic acid) can cross the blood-brain barrier and function in the brain for at least 1–12 h after oral YKS administration [27–30]. Thus, it is important to identify effective and metabolic components in YKS that are capable of crossing the blood-brain barrier.

## 5. Conclusion

In conclusion, our findings suggest that YKS downregulates miR-18 expression and normalizes HPA axis activity by recovering GR protein expression in the hypothalamus of stress-exposed mice. Elucidating the functional roles of the YKS-miR-18-GR protein-regulating pathway in the PVN of the hypothalamus is a primary goal of future research.
